# Effects of iron and boron combinations on the suppression of *Fusarium* wilt in banana

**DOI:** 10.1038/srep38944

**Published:** 2016-12-12

**Authors:** Xian Dong, Min Wang, Ning Ling, Qirong Shen, Shiwei Guo

**Affiliations:** 1 College of Pharmaceutical Science, Yunnan University of Traditional Chinese Medicine, Kunming 650500, China; 2 College of Resources and Environmental Science, Nanjing Agricultural University, Nanjing 210095, China

## Abstract

The effects of mineral nutrient on banana wilt disease, which are the result of a competitive relationship between host plants and pathogens, can affect the interactions of plants with microorganisms. To investigate the mineral nutrient effect, hydroponic experiments were conducted in glasshouse containing combinations of low, medium, and high iron (Fe) and boron (B) concentrations, followed by pathogen inoculation. High Fe and B treatment significantly reduced the disease index and facilitated plants growth. With increasing Fe and B concentrations, more Fe and B accumulated in plants. High Fe and B treatment dramatically reduced the *Fusarium oxysporum* conidial germination rate and fungal growth compared with the other two treatments, contributing to decreased numbers of the pathogen on infected plants. Furthermore, High Fe and B treatment decreased the fusaric acid production of *F. oxysporum in vitro* and also increased the mannitol content of the plants, which in turn decreased the phytotoxin production of infected plants and finally reduced the disease index due to the virulence factor of phytotoxin. Taken together, these results indicate that Fe and B play a multifunctional role in reducing the severity of diseases by affecting the growth of *F. oxysporum* and the responses between plants and pathogens.

*Fusarium* wilt of banana (Panama disease) caused by the pathogen *Fusarium oxysporum* f. sp. *cubense* (FOC) is a serious vascular disease. The pathogen is divided into four races based on differences in pathogenicity on Musa cultivars; race 4 is ranked as the most virulent race and can infect all Cavendish cultivars^1^. *Fusarium oxysporum* can survive for long periods in the absence of the host, mainly in the form of thick-walled chlamydospores. During the infection process, *F. oxysporum* can produce phytotoxins such as fusaric acid (FA; 5-*n*-butyl-pyridine-2-carboxylic acid, C_10_H_13_NO_2_)^2^, a well-known nonspecific toxin that disturbs the metabolism of the infected plant. It induces early hyperpolarization of plant cells followed by significant depolarization of transmembrane electrical potential and alters membrane permeability^3^. Evidence exists that FA plays a direct role in pathogenesis, and the concentration of FA detected in diseased plants is positively correlated with the virulence of *F. oxysporum*^4^. The initial symptom of banana wilt is water-soaked lesions on the leaves, followed by progressive leaf chlorosis from the lower leaves upward to the upper leaves. In cross sections of the pseudostem, a brown ring can be seen at the vascular bundle, indicating that fungal colonization is occurring^5^,^6^.

Management of *F. oxysporum* wilt is mainly performed through chemical soil fumigation and the use of resistant cultivars^6^. The most cost-effective, environmentally safe method of control is the use of resistant cultivars, when available^7^. However, breeding for resistance can be very difficult, especially when no dominant gene is known. In addition, new races of the pathogen can develop that have overcome host resistance^8^. The difficulty in controlling *F. oxysporum* wilt has stimulated research in nutritional control of *F. oxysporum* wilt^6^. Nutrients are important for the growth and development of both plants and microorganisms, which could affect the disease tolerance or resistance of plants to pathogens^9^. The disease resistance of the host is defined by its ability to limit the penetration, development, and reproduction of the invading pathogens. In contrast, the tolerance of the host is measured in terms of its ability to maintain its own growth or yield despite the infection^10^.

Iron (Fe) and boron (B) are two of the most important micronutrients for plant growth^11^. The role of Fe in plant pathogenesis caused by microbes is an emerging topic with exciting recent developments^12^. The effects of B on tobacco were studied because of the suggested connection between B nutrition and phenol metabolism^13^. Mannitol, a six carbon sugar alcohol, is the most widely distributed in nature and accumulates in response to abiotic stress as a compatible solute. Because sugar alcohols contain cis-hydroxyl groups, it can readily bind to boric acid and is likely to allow boron to be transported through phloem. Although the interactions between plants and pathogens have been well studied^5^,^14^, the roles of Fe and B in disease resistance and the dynamic interaction between plant nutrients and the plant–pathogen system are not well known. The present study was performed to identify safe measures to reduce the disease index of banana wilt and clarify the effect of Fe and B combinations on plant resistance.

## Results

### Effects of different Fe and B concentration on banana growth and disease index of banana wilt

After treatment with nutrient solution containing different Fe or B for 2 weeks, plants were inoculated with *F. oxysporum*. Fifteen days later, the disease severity was evaluated according to the standard disease scale. The results showed that the wilt disease index was almost the same among three different B concentration treatment. However, high B concentration enhanced banana growth (Table 1), whereas the high Fe concentration reduced the disease index (Table 1). It was observed that high Fe concentration could reduce the severity of wilt disease without growth promotion to banana seedlings; conversely high B concentration increased plant growth with little effect on the disease index (Table 1).

### Effects of Fe and B treatments on the disease index of banana wilt

**Figure 1:**
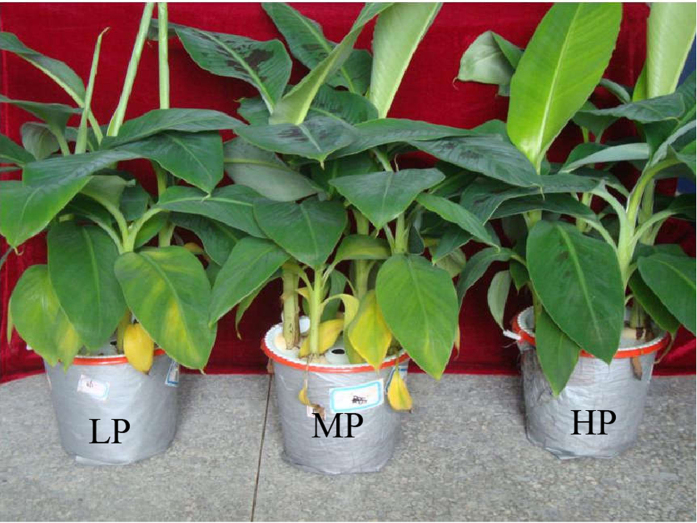
Programmed chlorosis of banana leaves after *Fusarium oxysporum* infection. Banana seedlings were treated with combinations of different concentrations of Fe and B. The six treatments were L, 0.5 ppm Fe and 0.05 ppm B-treated plants; M, 2 ppm Fe and 0.2 ppm B-treated plants; H, 20 ppm Fe and 2 ppm B-treated plants; LP, 0.5 ppm Fe and 0.05 ppm B-treated plants and *F. oxysporum* infection; MP, 2 ppm Fe and 0.2 ppm B-treated plants and *F. oxysporum* infection; and HP, 20 ppm Fe and 2 ppm B-treated plants and *F. oxysporum* infection.

After treatment with Fe and B for 2 weeks, the plants were inoculated with *F. oxysporum*. The results revealed that a combination of high concentrations of Fe and B (HP) significantly reduced the disease severity as compared to the low (LP) and middle (MP) concentrations (Fig. 1, Table 2). Many lower leaves of plants given the LP treatment showed serious chlorosis, as compared to the MP and HP treatments. The statistical results showed that the disease index of the LP, MP, and HP treatments were 55, 33.3, and 0, respectively (Table 2).

### Distribution of Fe and B in leaves and roots of banana seedlings

The Fe and B contents of tissues from plants subjected to the different treatments were determined with ICP-AES. The Fe contents in both leaves and roots were dramatically enhanced with treatments of increasing Fe concentrations, especially in the H treatment (Table 3). The most significant increases in the Fe content were observed with the H treatment in both leaves and roots, with increases of 1.90 and 8.74 times in leaves and roots, respectively, over those in the L treatment. After the FOC infection, the Fe content did not change in either the LP or the MP treatment relative to the L or M treatments, respectively, but an obvious reduction was observed in the HP treatment compared with the H treatment. The B content was also significantly greater in plants given the H treatment as compared to the L or M treatment. The leaf and root B contents of plants given the H treatment were 2.67 and 2.04 times higher, respectively, than those from plants given the L treatment. When the plants were infected, the B content remained high only in plants given the HP treatment; it was almost unchanged after infection, in contrast to plants given the LP and MP treatments.

### Effects of Fe and B treatments on banana seedling growth

To evaluate the effects of Fe and B treatments on the growth of banana seedlings, dry weight (DW) was measured (Table 4). The DW of leaves, pseudostem, and roots were significantly enhanced with a combination of increased Fe and B concentrations. The H treatment dramatically increased the biomass of all three tissues. The most significant increase in DW was observed in the H treatment, which resulted in DW increases of 1.68, 1.47, and 1.55 times in leaf, pseudostem, and root, respectively, over the L treatment. Following FOC infection, the DW of leaves, pseudostems, or roots was significantly different from that in noninfected plants.

### Leaf gas exchange of banana leaves

To compare the photosynthetic parameters of the treatment groups, the net photosynthetic rate (P_n_), stomatal conductance (g_s_), intercellular CO_2_ concentration (C_i_), and transpiration rate (T_r_) were measured (Table 5). These parameters did not change under the treatments with different Fe and B combinations, but after FOC infection, reductions in some of these parameters were observed in the LP, MP, and HP treatments. The most significant decrease in the net photosynthetic rate (P_n_) was observed in the LP treatment, with 2.76-, 1.67-, and 1.21-fold decreases in the LP, MP, and HP treatments compared with the L, M, and H treatments, respectively.

### *F. oxysporum* numbers in banana seedlings under different Fe and B concentrations

**Figure 2:**
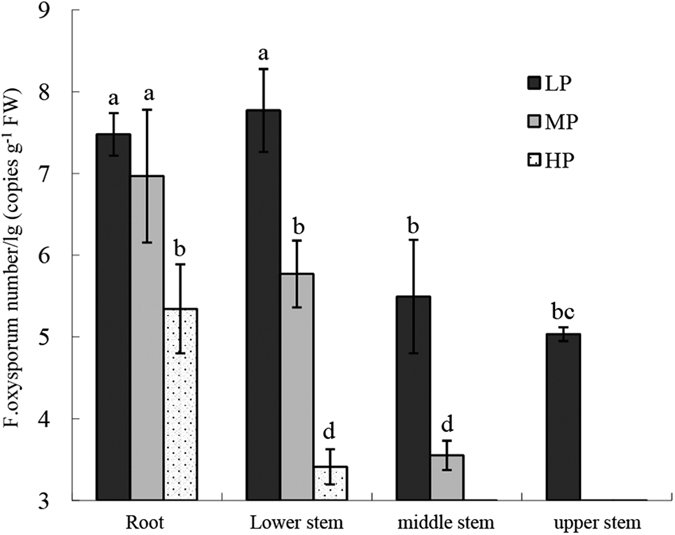
Quantitative detection of *Fusarium oxysporum* f. sp. *cubense* in inoculated banana seedlings. Three different treatments were LP, 0.5 ppm Fe and 0.05 ppm B-treated plants with *F. oxysporum* infection; MP, 2 ppm Fe and 0.2 ppm B-treated plants with *F. oxysporum* infection; and HP, 20 ppm Fe and 2 ppm B-treated plants with *F. oxysporum* infection. The infected plants were divided into four parts from the root upward to the upper pseudostem. The results represent means ± SD of four replicates.

Quantitative detection of *F. oxysporum* f. sp. *cubense* showed that the pathogen was mainly present in the root and lower stems (Fig. 2). The pathogen numbers in these two parts were significantly higher than in other parts of the plant, suggesting that the pathogen spread from the root upward to the shoot. The highest pathogen numbers were observed in the root and lower stem of low concentration-treated plants, which were 7.5 and 7.8 lg (copies g^−1^ FW), respectively (LP). In medium concentration-treated plants, the highest pathogen numbers in the root and lower stem were 6.9 and 5.8 lg (copies g^−1^ FW), respectively (MP). In high concentration treated-plants, the pathogen was only detected in the root and lower stem, and the pathogen numbers in these two tissues were significantly lower than with the other two treatments: 5.3 and 3.4 lg (copies g^−1^ FW), or only 70% and 43% of the numbers in low concentration-treated plants (HP).

### Effects of Fe and B on the conidial germination rate, mycelium growth, and FA production

**Figure 3:**
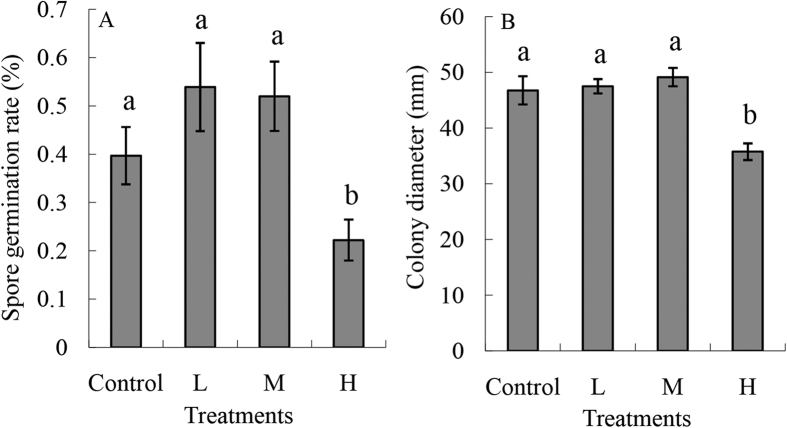
(**A**) *Fusarium oxysporum* spore germination rates in plants given nutrient solutions with different Fe and B concentrations. The four treatments were Control, 0 ppm Fe and 0 ppm B in Hoagland nutrient solution; L, 0.5 ppm Fe and 0.05 ppm B in Hoagland nutrient solution; M, 2 ppm Fe and 0.2 ppm B in Hoagland nutrient solution; and H, 20 ppm Fe and 2 ppm B in Hoagland nutrient solution. (**B**) Mycelium growth of *F. oxysporum* in agar plates with different Fe and B concentrations. The four treatments were control, 0 ppm Fe and 0 ppm B in Hoagland nutrient solution in 2% water agar; L, 0.5 ppm Fe and 0.05 ppm B in Hoagland nutrient solution in 2% water agar; M, 2 ppm Fe and 0.2 ppm B in Hoagland nutrient solution in 2% water agar; H, 20 ppm Fe and 2 ppm B in Hoagland nutrient solution in 2% water agar. The Hoagland nutrient solution is described in the Materials and methods. Different small letters in the same group indicate a significant statistical difference according to the two-way analysis of variance (*P* < 0.05).

To determine the effects of Fe and B on the germination rate, nutrient solutions with different concentrations were prepared. High concentrations of Fe and B (H) dramatically reduced the *F. oxysporum* conidial germination rate as compared to the other three treatments (Control, L, and M). The conidial germination rate in the H treatment was about 40% lower than in the L or M treatments (Fig. 3A).

The fungal mycelium growth in agar plates containing high Fe and B was lower than in the other three treatments (Control, L, and M) after 6 days. The *Fusarium* colony diameter in H treatment was the smallest among the four treatments, and the other three treatments did not show a significant difference (Fig. 3B).

**Figure 4:**
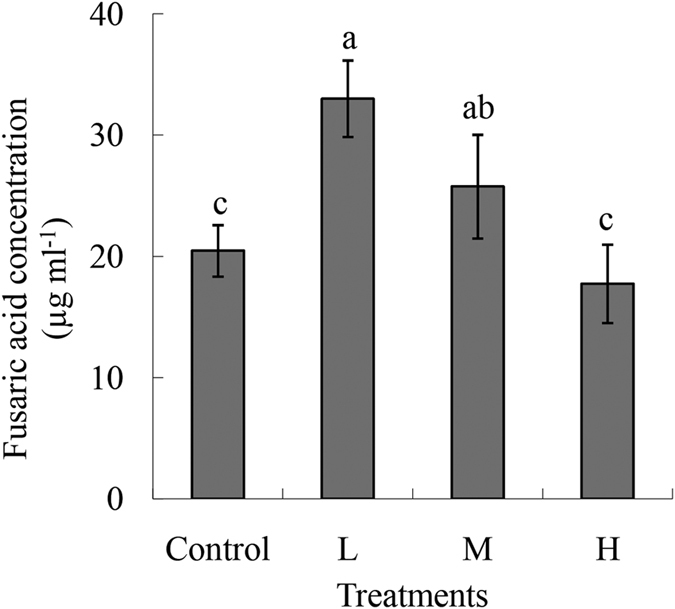
Production of fusaric acid by *Fusarium oxysporum* grown under four different nutrient conditions. The conditions were Control, *F. oxysporum* cultured in Czapek Dox medium; L, *F. oxysporum* cultured in Czapek Dox medium supplemented with 0.5 ppm Fe and 0.05 ppm B; M, *F. oxysporum* cultured in Czapek Dox medium supplemented with 2 ppm Fe and 0.2 ppm B; and H, *F. oxysporum* cultured in Czapek Dox medium supplemented with 20 ppm Fe and 2 ppm B. Different small letters indicate a significant statistical difference according to the two-way analysis of variance (*P* < 0.05).

The FA production was the highest with the L treatment. Compared with the other two treatments (Control and H), the FA production in L and M were 1.60-fold and 1.30-fold higher, respectively. No significant difference in FA production was observed between the control and H treatments (Fig. 4).

### Effects of *F. oxysporum* infection on the mannitol contents of banana leaves and roots

**Figure 5:**
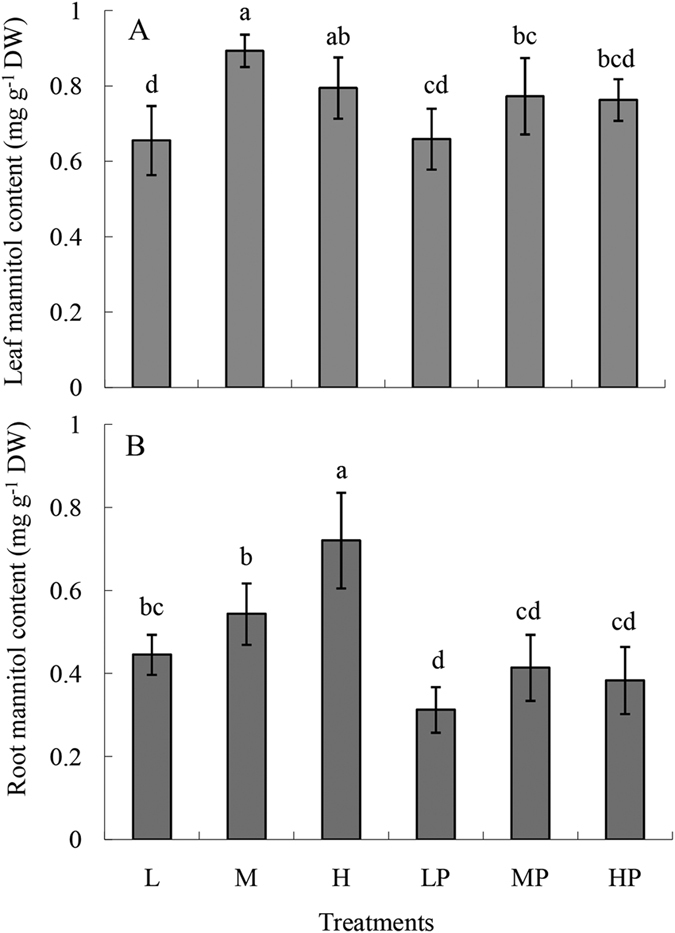
Mannitol contents of leaves (**A**) and roots (**B**) from plants grown under treatments with different concentrations of Fe and B. The six treatments were as described in Fig. 1. The results shown are means ± SD of four replicates. Different small letters indicate a significant statistical difference according to the two-way analysis of variance (*P* < 0.05).

Plants treated with different combinations of Fe and B concentrations were found to contain different mannitol content in both leaves and roots. The mannitol contents increased with increasing concentrations of Fe and B (Fig. 5A,B). In banana leaves, the mannitol contents were 1.3 and 1.22 times higher in the M and H treatments, respectively, than in the L treatment. Roots showed 1.22- and 1.62-fold increases in the M and H treatments, respectively, over the L treatment. When plants were infected, the leaf mannitol content was relatively stable, whereas in roots, the mannitol content was dramatically lower with all treatments, decreasing to 70%, 76%, and 53%, respectively, that of non-infected plants (Fig. 5A,B).

### Effect of mannitol content on FA production

**Figure 6:**
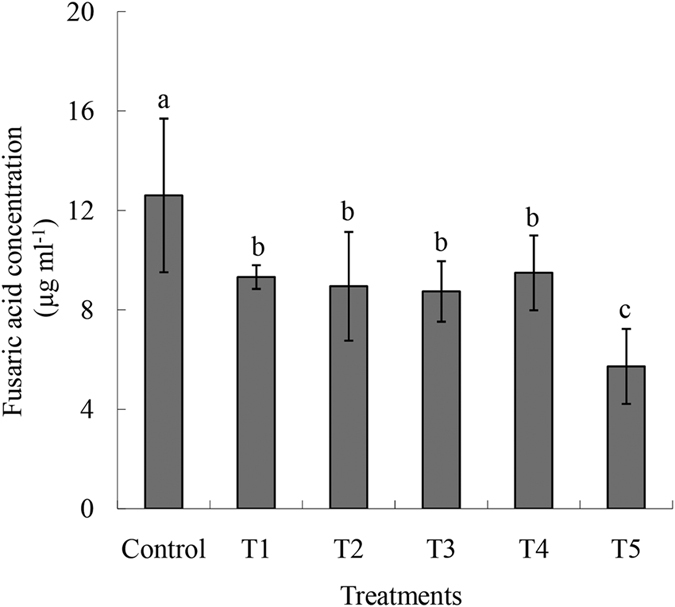
Production of fusaric acid in Czapek Dox medium in which the sucrose ingredient was replaced by mannitol in different ratios. The six treatments were control, the original sucrose composition of Czapek Dox medium (30 mg·ml^−1^); T_1_, 25 mg·ml^−1^ sucrose and 5 mg·ml^−1^ mannitol in Czapek Dox medium; T_2_, 20 mg·ml^−1^ sucrose and 10 mg·ml^−1^ mannitol in Czapek Dox medium; T_3_, 15 mg·ml^−1^ sucrose and 15 mg·ml^−1^ mannitol in Czapek Dox medium; T_4_, 10 mg·ml^−1^ sucrose and 20 mg·ml^−1^ mannitol in Czapek Dox medium; and T_5_, 5 mg·ml^−1^ sucrose and 25 mg·ml^−1^ mannitol in Czapek Dox medium. Different small letters indicate a significant statistical difference according to the two-way analysis of variance (*P* < 0.05). The English in this document has been checked by at least two professional editors, both native speakers of English. For a certificate, please see: http://www.textcheck.com/certificate/MgddWy.

To illustrate the effects of mannitol on the production of FA, the sugar ingredient in Czapek Dox medium was replaced by mannitol in different ratios. The results showed that FA production decreased with increasing concentrations of mannitol and decreasing concentrations of sugar compared with the control, particularly when the mannitol concentration was 25 mg ml^−1^. At this concentration, the production of FA was 55% lower than in the control treatment (Fig. 6).

### FA content of banana seedlings after *F. oxysporum* infection

FA produced by *Fusarium* species was detected in all tested tissues of infected banana plants (Table 2). The concentrations of FA in roots, pseudostem, and leaves of plants given the LP treatment were 1.42 μg g^−1^, 1.11 μg g^−1^, and 8.64 μg g^−1^, respectively. The FA concentrations in three different organs of plants given the HP treatment were dramatically lower than in corresponding organs of plants given the LP treatment: 0.13 μg g^−1^, 0.28 μg g^−1^, and 1.39 μg g^−1^, respectively, or only 16%, 25%, and 9.3% of the LP treatment, respectively.

## Discussion

Plant diseases continue to play a major limiting role in agricultural production. The control of plant diseases using classical pesticides raises serious concerns about food safety, environmental quality and pesticide resistance, which have dictated the need for alternative disease management way^15^. Nutrients are important for growth and development of plants and also microorganisms, and they are important factors in disease control^16^. All the essential nutrients can affect disease severity^15^,^17^. The effects of micronutrients on reducing the severity of diseases can be attributed to their involvement in physiology and biochemistry of the plant, as many of the essential micronutrients are involved in many processes that can affect the responses of plants to pathogens^11^. Fe and B are micronutrients of plants, but their role in microbial plant pathogenesis is not well understood.

Our preliminary experiment was to determine the different B concentration on wilt disease. The results showed that different concentration of B did not influence the wilt disease index. However, high B concentration significantly promoted the growth of both banana root and shoot (Table 1). The effect of Fe on banana wilt disease was also tested. It was observed that high Fe concentration could reduce the severity of wilt disease without growth promotion to banana seedlings (Table 1). This result was the same with Peng’s result^18^. They found that through amending the soils with iron chelate could reduce the germination of chlamydospores and disease severity in banana plantlets. Therefore, we applied Fe and B combination to try to get the purpose of both growth and resistance enhancement to banana seedlings.

Through analysis of the Fe and B distribution in plants, we found that the Fe and B contents in both leaf and root were dramatically enhanced with increasing Fe and B concentrations, especially in the H treatment (Table 3). To examine different Fe and B concentration combinations on the growth of banana plants, we measured the dry weight and photosynthetic parameters (P_n_, g_s_, C_i_, T_r_). The results showed that high applications dramatically increased the biomass of all three tissues (Table 4). However, photosynthetic parameters did not change under the treatments with different Fe and B combinations (Table 5). Following FOC infection, a strong reduction in the Fe content was observed in the HP treatment, and the B content remained at a relatively high level. Fe plays a crucial role in redox systems in cells and in various enzymes, and B is crucial for cell wall and membrane integrity^19^. *Fusarium* infection could disturb the water balance of infected plant, due to damage of cell membrane^20^. B promotes stability and rigidity of the cell wall structure possibly is involved in the integrity of the plasma membrane^15^. A high B content in leaves after *F. oxysporum* infection could help maintain the integrity of the membrane and cell wall, which was one of the important requirements of the high tolerance induced by the H treatment (Table 2). The availability of Fe for both plants and pathogen may be quite low. Fe deficiency results in increased sensitivity to symptom development. Supplementation of plants with Fe significantly alleviated disease symptoms^21^. Similar results were obtained by Singh and Khanna^14^, who found that Fe and B deficiencies both contribute to increased lesion size, and that the low availability of these nutrients affected the accumulation of lignin. Furthermore, in the case of *Dickeya dadantii*, Fe deficiency caused a reduction in bacterial fitness and expression of virulence genes as well as an exacerbation of the salicylic acid-mediated defense pathway^22^. Through adding Fe-EDDHA to the soil, it was increased suppressiveness to *Fusarium oxysporum* f. sp. *cubense*^18^. Thus, the plant Fe status could influence host–pathogen relationships in different ways by affecting the pathogen’s virulence as well as the host’s defense^19^,^22^. In the present study, we observed that the conidial germination rate and fungal mycelium growth were greatly reduced by treatment with a high -Fe and -B nutrition solution (Fig. 3). Similar results were obtained by Singh and Khanna^23^, who showed that fungal conidia were very sensitive to even low amounts of Fe and B. Low amounts of Fe and B in the medium stimulated fungal growth and conidial germination, and a significant reduction in growth was observed when the nutrient concentrations were higher than 20 ppm. Reduced conidial germination and fungal mycelium growth rate would be expected to result in a decrease in the pathogen inoculum infecting the plant (Fig. 2). The reduced pathogen levels in H treatment could alleviate the injury of infected plant, which in turn maintain the relatively high photosynthetic capacity (Table 5).

FA is a nonspecific wilt-inducing toxin produced by *F. oxysporum* species, and the pathogenicity of this fungus is positively correlated with the FA content^24^,^25^. Conditions favorable for *in vitro* growth are also generally the most favorable for mycotoxin production^26^. Our study has shown that the toxin production was strongly reduced when the concentrations of Fe and B in the solution were 20 ppm and 2 ppm, respectively (Fig. 4).

B is not efficiently remobilized in many plant species, and sugar alcohols produced by plants, including mannitol and sorbitol, are mainly responsible for the phloem translocation of boric acid^27^,^28^. In our study, the mannitol contents of both leaves and roots were higher in the H treatment than in the L or M treatments (Fig. 5). The increased mannitol content was likely the reason that B was present at high levels in both leaves and roots in the H treatment (Table 3).

What’s the effect of increased mannitol of plant on *Fusarium*? In Son’s study, when mannitol supplement in medium, conidia was converted to chlamydospore and several genes are involved in this conidial modification. Fungi use mannitol to store carbon, balance redox and serve as an antioxidant^29^. Therefore, in addition to the role in B transport and increasing the resistance to both biotic and abiotic stresses^30^, mannitol could also be used as an energy source by *F. oxysporum*. FA plays a critical role in accelerating the development of Fusarium wilt in banana plants by acting as a phytotoxin^20^. To examine the toxin production of *Fusarium* on increased mannitol level, we set a series of medium containing different mannitol concentration. It was shown that both the higher concentration of mannitol and the lower concentration of sugars compared with the original concentration of culture medium probably helped decrease toxin production (Fig. 6). Although mannitol treatment could reduce the disease severity of tomato wilt^31^, the function of mannitol in the plant–pathogen interaction was not clear.

Fusarium wilt diseases are the result of the interaction between a host plant and a pathogen^6^. For *F. oxysporum*, entering the host and attaching to target tissues are tightly controlled by the production of virulence factors that promote the establishment of the microbe and the evasion of host defense^32^. Infection with *F. oxysporum* results in injury to the membrane system, which is a major effect of this disease. The injury is caused by the FA produced by the pathogen^20^. To produce disease, plant-pathogenic fungi must be able to grow on the host tissue. During root invasion and colonization, *F. oxysporum* is exposed to various plant defense mechanisms, such as physical barriers and antifungal compounds^33^,^34^. After penetration, the next step in a fungal strategy to colonize a plant species is often the secretion of toxins or plant hormonelike compounds that manipulate the plant’s physiology to the benefit of the pathogen^32^. In the present study, the number of *F. oxysporum* in infected plants was significantly lower in the treatment with high concentrations of Fe and B (HP), as compared to the LP and MP treatments (Fig. 2). The decreased numbers of the pathogen contributed to the reduced FA production in infected plants, which resulted in a lower disease index (Fig. 2, Table 1).

## Conclusion

Plants treated with combinations of a high content of Fe and B show increased resistance to *F. oxysporum* infection. High Fe and B contents in the plant correlated with decreased conidial germination rate, fungal growth, and FA production. The increased mannitol content may have affected the interaction between the plant and the pathogen, which in turn reduced the levels of FA produced by *F. oxysporum* in infected plants. Thus, it may be useful to take into account the plant Fe and B status when a need exists to control disease without compromising crop quality and yield in economically important plant species.

## Methods

### Plant Cultivation

Banana seedlings (cv. Gross Michel, *Musa* spp. AAA group) highly susceptible to *F. oxysporum* f. sp. *cubense* race 4 [*F. oxysporum* Schlecht. f. sp. *cubense* (E.F. Smith) Snyd. and Hans.] were obtained from a local banana production site in Hainan Province, China. After 4 weeks of pre-culture in a growth chamber, the seedlings were transplanted into 2.5-L barrels with half-strength Hoagland’s nutrient solution and grown under a day/night temperature cycle of 30 °C/25 °C with a relative humidity of 70% ± 10% and a photoperiod of 14 h day^−1^ (photosynthetic photon flux density >300 μmol m^−2^ s^−1^). Two weeks later, the seedlings were supplied with full-strength Hoagland nutrient solution. The macronutrient composition of the Hoagland nutrient solution (in mg l^−1^) was 40 N (as NH_4_NO_3_), 10 P (as KH_2_PO_4_), 40 K (as K_2_SO_4_ and KH_2_PO_4_), 57 Ca (as CaCl_2_), and 40 Mg (as MgSO_4_). The basal micronutrient composition (in mg·l^−1^) was 2.0 Fe (as Fe–EDTA), 0.2 B (as H_3_BO_3_), 0.5 Mn (as MnCl_2_·4H_2_O), 0.05 Mo [as (NH_4_)_6_Mo_7_O_24_·4H_2_O], 0.01 Zn (as ZnSO_4_·7H_2_O), and 0.01 Cu (as CuSO_4_·5H_2_O).

When the plants grew to the 5 to 6-leaf stage, the seedlings were treated with Hoagland nutrient solutions containing different contents of Fe and B. Fe–EDTA and H_3_BO_3_ were used as the Fe and B sources, respectively. The concentrations were classified into three levels: low, 0.5 ppm Fe + 0.05 ppm B; medium, 2 ppm Fe + 0.2 ppm B; and high, 20 ppm Fe + 2 ppm B. Each treatment group consisted of 60 plants. The placement of plants in the greenhouse was randomized to avoid edge effects.

### Preparation of fungal cultures

FOC was cultured on potato dextrose agar (PDA) medium at 28 °C in darkness for 7 days. Then, 8-mm-diameter discs of fungus-containing agar were excised from the culture margins and inoculated into 500-ml Erlenmeyer flasks containing Bilay’s medium^4^. The flasks were incubated for 6 days at 28 °C with rotary shaking at 180 rpm. The resulting fungal cultures were filtered through four layers of cheesecloth to remove the mycelia and then centrifuged at 8,000× *g* for 20 min to pellet the conidia. The conidia were resuspended in sterile water and quantified using a hemocytometer.

### Inoculation of plants with conidia

After the banana seedlings had grown for 14 days in solutions containing different Fe and B concentrations, they were carefully removed from the nutrient solution. The roots were submerged for 2 h in a conidial suspension containing 4 × 10^6^ spores ml^−1^, and the plants were then transplanted into a barrel with a nutrient solution containing 5 ml of conidial suspension to ensure infection. Control plants were treated similarly except that sterile water was used instead of the conidial suspension.

### Disease severity

After 15 days of infection by *F. oxysporum*, the plants were graded for severity of Fusarium wilt disease as 0 (not showing chlorosis), 1 (one or two lowest leaves showing chlorosis), or 2 (three or four lowest leaves showing chlorosis). The percent disease index was calculated as follows: disease index = ∑ (rating × number of plants rated)/(total number of plants × highest rating) × 100^35^. All of the measurements below were implemented after 15 days of infection by *F. oxysporum.*

### Assessment of Fe and B contents of banana plants

Prior to analysis, samples were homogenized and prepared for inductively coupled plasma-atomic emission spectroscopy (ICP-AES) analysis. First, 0.500 g of leaf and root tissues of banana plants was accurately weighed into an acid-washed Teflon digestion tube. Then 8 ml of 16 mol l^−1^ HNO_3_ and 2 ml of 12.38 mol l^−1^ HClO_4_ were added, and the tube was heated in a microwave oven until the solution was clear. To measure the Fe and B concentrations in leaf and root material, the solutions were analyzed using ICP-AES^36^.

### Assessment of fresh weight and dry weight of banana plants

The fresh weights of leaf, pseudostem, and root tissues were determined at the time of harvest. Fresh samples were heated to 105 °C for 30 min and then dried at 80 °C to constant weight to determine the dry weight.

### Gas exchange measurements

Net photosynthetic rate (P_n_), transpiration rate (T_r_), and stomatal conductance (g_s_) were measured at 28 °C using a portable photosynthesis system (LI-6400; Li-Cor Biosciences, Lincoln, NE). During these measurements, the leaves were maintained at a temperature of 28 °C, a relative humidity of 50%, and a photosynthetic photon flux density of 1,000 μmol photons m^−2^ s^−1^. Data were recorded after the systems reached a steady-state equilibrium (approximately 10 min).

### Determination of the conidial germination rate of *F. oxysporum*

Hoagland nutrient solutions containing various concentrations of Fe and B were prepared. The concentrations were classified into four levels: Control, 0 ppm Fe + 0 ppm B; low, 0.5 ppm Fe + 0.05 ppm B; medium, 2 ppm Fe + 0.2 ppm B; and high, 20 ppm Fe + 2 ppm B. The fungal conidial suspension was obtained from 7-day-old cultures of *F. oxysporum* as described by Hao *et al*.^37^ with slight modifications, and the number of spores was adjusted to 2 × 10^6^ spores per milliliter. The conidial germination rate was measured as described by^38^ with some modifications. Fifty microliters of fungal conidial suspension was mixed with 0.5 ml of nutrient solution or sterile distilled water. Then 100 μl of the mixture was then placed on separate concave glass slides. The slides were incubated for 24 h at 28 °C in darkness in a plastic container lined with moist tissue paper. The conidial germination rates were determined by microscopic observation. The percent conidial germination rate was calculated using the following formula: spore germination rate (%) = (the number of spores germinated/the number of spores observed) × 100.

### Measurement of *F. oxysporum* mycelium growth in agar plates

The effects of Fe and B on *F. oxysporum* growth were determined by growing the fungus on plates containing different amounts of Fe and B. Varying concentrations of these nutrients were added to 2% (w/v) water agar before it had solidified in standard 90-mm Petri dishes containing a total volume of 20 ml, according to the method of^37^ with slight modifications. The final concentrations of Fe and B in the agar plates were 0 ppm Fe and 0 ppm B (Control), 0.5 ppm Fe and 0.05 ppm B (Low), 2 ppm Fe and 0.2 ppm B (Medium), and 20 ppm Fe and 2 ppm B (High). The undersides of the colonies grown on the agar plates were measured with a ruler to determine the rate of fungal growth at various times^39^.

### Assessment of mannitol content in plant tissues

To determine the mannitol content in plant tissues, dry powder (0.5 g) was extracted twice with 10 ml of boiling distilled water for 2 h. The resulting filtrate was collected and adjusted to a final volume of 25 ml. One milliliter of the solution containing the extract was mixed with 1 ml of sodium periodate (0.015 mol l^−1^). After 10 min, 2 ml of rhamnose (0.1%) and 4 ml of fresh Nash reagent (2 mol l^−1^ ammonium acetate mixed with 2 ml acetic acid and 2 ml acetyl acetone) were added to the mixture, which was then placed in a water bath at 53 °C for 15 min. A blank sample was prepared by substituting distilled water for the extract solution. The absorbance at 412 nm was measured on spectrophotometer (T6; Beijing Purkinje General Instrument Co., Ltd., Beijing, China). A standard curve was prepared using a mannitol standard. One milliliter of solution containing up to 50 μg ml^−1^ of mannitol was determined by the above method, and the mannitol content of samples was calculated from a linear regression equation created from the standard curve^40^.

### Extraction of FA from banana seedlings

Banana leaf, pseudostem, and root were harvested separately after the plant had been inoculated with FOC for 15 days, and then washed in tap water and wiped dry with filter paper. The tissues were weighed and homogenized in a juice extractor with MeOH/1% KH_2_PO_4_ (1:1, v/v, pH 2.5). The suspension was then centrifuged at 10,000× *g* for 15 min. The clarified supernatants were pooled and the pH of the supernatant was adjusted to 2.5 with 2 M HCl. The acidified supernatant was sequentially extracted with 50 ml of methylene chloride. The methylene chloride extracts were pooled and evaporated to dryness at 45 °C on a rotary evaporator. The residue was redissolved in 3 ml of MeOH and stored at −20 °C until analysis by high-performance liquid chromatography (HPLC) as described by^20^.

### Specific detection of *F. oxysporum* by real-time polymerase chain reaction (PCR)

Extraction of DNA from infected banana seedlings was performed according to^41^ by grinding 100 mg of four plant tissues (root, lower stem, middle stem, upper stem) in a mortar with liquid nitrogen. The isolated DNA samples were then used as templates for PCRs. The *F. oxysporum* f. sp. *cubense*-specific primers (5′-CAGGGGATGTATGAGGAGGCT and 5′-GTGACAGCGTCGTCTAGTTCC) were used in a real-time PCR assay^41^. Real-time PCR amplification was performed in 25-μl reaction mixtures containing 12.5 μl SYBR Green PCR Master Mix (Takara, Dalian, China), 0.5 μl ROX dye (50×), 0.5 μM of each primer, and 1 μl of template DNA. The PCR program was 94 °C for 3 min, followed by 29 amplification cycles at 94 °C for 45 s, 58 °C for 45 s, and at 72 °C for 1 min. To evaluate the amplification specificity, melt-curve analysis was performed at the end of the PCR run. Standard curves were generated according to a previous report and the abundances of FOC were expressed as copy concentration as described previously^42^.

### Extraction of FA from pathogen culture media containing different contents of mannitol and combinations of Fe and B

To determine the effects of Fe and B on FA production, FOC was inoculated into Czapek Dox medium (40 ml in 100-ml flasks) amended with Fe–EDTA and H_3_BO_3._ Four different concentrations of Fe and B culture medium were prepared. The treatments were 0 ppm Fe and 0 ppm B (Control), 0.5 ppm Fe and 0.05 ppm B (Low), 2 ppm Fe and 0.2 ppm B (Medium), and 20 ppm Fe and 2 ppm B (High). The pathogen was incubated at 28 °C on a rotary shaker (180 rpm) for about 10 days^4^. The culture was filtered with a 0.45-μm membrane to exclude mycelia and microconidia. Subsequently, the filtrate was adjusted to pH 2.5 with 2 M HCl and extracted three times with an equal volume of methylene chloride. The organic phase (methylene chloride) was pooled and lyophilized under a vacuum. The residue was dissolved in 3 ml of methanol to obtain the crude toxin solution, and then the concentration of FA was determined by HPLC as described by^20^.

To determine the effect of mannitol on FA production, FOC was inoculated in Czapek Dox medium (40 ml in 100-ml flasks) and the sugar ingredient was replaced by mannitol in different ratios. Six treatments were performed: Control, the original sugar composition of Czapek Dox medium (30 mg ml^−1^); T_1_, 25 mg ml^−1^ sugar and 5 mg ml^−1^ mannitol in Czapek Dox medium; T_2_, 20 mg ml^−1^ sugar and 10 mg ml^−1^ mannitol in Czapek Dox medium; T_3_, 15 mg ml^−1^ sugar and 15 mg ml^−1^ mannitol in Czapek Dox medium; T_4_, 10 mg ml^−1^ sugar and 20 mg ml^−1^ mannitol in Czapek Dox medium; and T_5_, 5 mg ml^−1^ sugar and 25 mg ml^−1^ mannitol in Czapek Dox medium. FA extraction and analysis were performed as described above.

### Statistical analysis

Statistical analysis was performed using the Statistix 9.0 software (Analytical Software, Tallahassee, Florida, USA). Differences between treatments were determined by the two-way analysis of variance, and *P* < 0.05 was taken to indicate statistical significance.

## Additional Information

**How to cite this article**: Dong, X. *et al*. Effects of iron and boron combinations on the suppression of *Fusarium* wilt in banana. *Sci. Rep.* **6**, 38944; doi: 10.1038/srep38944 (2016).

**Publisher's note:** Springer Nature remains neutral with regard to jurisdictional claims in published maps and institutional affiliations.

## Figures and Tables

**Table 1:** Disease index and dry weight of banana seedlings under different B and Fe concentration with *F. oxysporum* infection.

	Disease index	Dry weight (g)	
		Leaf	Root
0.05 ppm B + 2 ppm Fe	45a	0.87 ± 0.03bc	0.26 ± 0.02b
2 ppm B + 2 ppm Fe	43a	1.16 ± 0.17a	0.33 ± 0.01a
0.2 ppm B + 2ppm Fe	41a	0.91 ± 0.09b	0.27 ± 0.03b
0.5 ppm Fe + 0.2 ppm B	48a	0.89 ± 0.01b	0.26 ± 0.03b
20 ppm Fe + 0.2 ppm B	8b	0.88 ± 0.03bc	0.24 ± 0.05bc

Other nutrients were the same with the Hoagland nutrient solution. Disease index = ∑(rating × number of plants rated)/(Total number of plants × highest rating) × 100.

**Table 2:** Disease index and fusaric acid contents of banana seedlings under different treatments.

	Disease index	Fusaric acid content (μg g^−1^ FW)		
		Leaf	Pseudostem	Root
LP	55a	8.64 ± 0.97a	1.11 ± 0.12a	1.42 ± 0.24a
MP	33.3b	5.41 ± 1.59b	0.61 ± 0.27b	0.41 ± 0.13b
HP	0c	1.39 ± 0.94c	0.28 ± 0.04c	0.13 ± 0.08c

The three different treatments were LP, 0.5 ppm Fe and 0.05 ppm B-treated plants with *F. oxysporum* infection; MP, 2 ppm Fe and 0.2 ppm B-treated plants with *F. oxysporum* infection; and HP, 20 ppm Fe and 2 ppm B-treated plants with *F. oxysporum* infection.

**Table 3:** Fe and B concentrations in leaves and roots in plants treated with different concentrations of Fe and B.

Treatments	Leaf (μg g^−1^ DW)		Root (μg g^−1^ DW)	
	Fe	B	Fe	B
L	78.41 ± 4.23d	13.98 ± 3.29bc	417.06 ± 58.78e	11.30 ± 1.11b
M	111.65 ± 9.20b	15.54 ± 1.45b	1178.98 ± 228.65 cd	10.19 ± 0.75b
H	149.00 ± 17.44a	37.35 ± 3.76a	3646.86 ± 414.26a	23.04 ± 2.75a
LP	88.89 ± 12.97 cd	10.64 ± 1.51 cd	814.47 ± 66.19e	8.57 ± 1.03b
MP	101.68 ± 7.76bc	9.88 ± 1.39d	1382.83 ± 132.43c	9.75 ± 0.76b
HP	105.69 ± 20.09bc	34.16 ± 2.83a	2950.46 ± 508.41b	20.38 ± 3.67a

The six treatments were L, 0.5 ppm Fe and 0.05 ppm B-treated plants; M, 2 ppm Fe and 0.2 ppm B-treated plants; H, 20 ppm Fe and 2 ppm B-treated plants; LP, 0.5 ppm Fe and 0.05 ppm B-treated plants and *F. oxysporum* infection; MP, 2 ppm Fe and 0.2 ppm B-treated plants and *F. oxysporum* infection; and HP, 20 ppm Fe and 2 ppm B-treated plants and *F. oxysporum* infection. The results shown are means ± SD of four replicates. Different small letters in the same column indicate a significant statistical difference according to the two-way analysis of variance (*P* < 0.05).

**Table 4:** The dry weight (DW) of leaves, pseudostems, and roots of plants under different treatments.

Treatments	DW (g plant^−1^)		
	Leaf	Pseudostem	Root
L	2.31 ± 0.58bc	1.09 ± 0.33b	0.78 ± 0.12b
M	2.99 ± 0.79b	1.19 ± 0.33b	0.87 ± 0.28b
H	3.88 ± 0.61a	1.60 ± 0.36a	1.21 ± 0.15a
LP	2.16 ± 0.22c	0.98 ± 0.09b	0.52 ± 0.07c
MP	2.79 ± 0.51bc	1.15 ± 0.11b	0.81 ± 0.19b
HP	3.81 ± 0.52a	1.56 ± 0.17a	1.23 ± 0.22a

The six treatments were as described in Table 2. The results shown are means ± SD of four replicates. Different small letters in the same column indicate a significant statistical difference according to the two-way analysis of variance (*P* < 0.05).

**Table 5:** Net photosynthetic rate (P_n_), stomatal conductance (g_s_), intercellular CO_2_ concentration (C_i_), and transpiration rate (Tr) of plants under different treatments.

Treatments	P_n_ (μmol m^−2^ s^−1^)	g_s_ (mmol m^−2^ s^−1^)	C_i_ (μmol mol^−1^)	Tr (mmol m^−2^ s^−1^)
L	14.83 ± 1.86a	0.195 ± 0.028a	363 ± 10.24a	4.095 ± 0.269a
M	16.6 ± 0.62a	0.197 ± 0.020a	351 ± 5.72ab	4.165 ± 0.366a
H	15.15 ± 0.69a	0.214 ± 0.048a	351 ± 11.56ab	4.493 ± 0.915a
LP	5.38 ± 2.08d	0.036 ± 0.019c	237 ± 59.88d	0.996 ± 0.467c
MP	9.94 ± 2.14c	0.072 ± 0.027bc	258 ± 57.04 cd	1.918 ± 0.581b
HP	12.45 ± 1.28b	0.112 ± 0.039b	302 ± 37.05bc	2.773 ± 0.696b

The six treatments were as described in Table 2. The results shown are means ± SD of four replicates. Different small letters in the same column indicate a significant statistical difference according to the two-way analysis of variance (*P* < 0.05).
